# Establishing and validating a pathway prognostic signature in pancreatic cancer based on miRNA and mRNA sets using GSVA

**DOI:** 10.18632/aging.103965

**Published:** 2020-11-10

**Authors:** Junfeng Zhang, Jianyou Gu, Shixiang Guo, Wenjie Huang, Yao Zheng, Xianxing Wang, Tao Zhang, Weibo Zhao, Bing Ni, Yingfang Fan, Huaizhi Wang

**Affiliations:** 1 Institute of Hepatopancreatobiliary Surgery, Chongqing General Hospital, University of Chinese Academy of Sciences, Chongqing 401120, P R China; 2 Department of Hepatobiliary Surgery, Zhujiang Hospital, Southern Medical University, Guangzhou 510280, Guangdong Province, P R China; 3 PLA Strategic Support Force Characteristic Medical Center (The 306th Hospital of PLA), Beijing 100101, P R China; 4 Department of Pathophysiology, College of High Altitude Military Medicine, Third Military Medical University, Chongqing 400038, P R China; 5 Key Laboratory of Extreme Environmental Medicine, Ministry of Education of China, Chongqing 400038, P R China; 6 Key Laboratory of High Altitude Medicine, PLA, Chongqing 400038, P R China

**Keywords:** pancreatic cancer, prognostic signature, miRNA sets, GSVA, metabolic pathways

## Abstract

Pancreatic cancer (PC) is a severe disease with the highest mortality rate among various cancers. It is urgent to find an effective and accurate way to predict the survival of PC patients. Gene set variation analysis (GSVA) was used to establish and validate a miRNA set-based pathway prognostic signature for PC (miPPSPC) and a mRNA set-based pathway prognostic signature for PC (mPPSPC) in independent datasets. An optimized miPPSPC was constructed by combining clinical parameters. The miPPSPC, optimized miPPSPC and mPPSPC were established and validated to predict the survival of PC patients and showed excellent predictive ability. Four metabolic pathways and one oxidative stress pathway were identified in the miPPSPC, whereas linoleic acid metabolism and the pentose phosphate pathway were identified in the mPPSPC. Key factors of the pentose phosphate pathway and linoleic acid metabolism, G6PD and CYP2C8/9/18/19, respectively, are related to the survival of PC patients according to our tissue microarray. Thus, the miPPSPC, optimized miPPSPC and mPPSPC can predict the survival of PC patients efficiently and precisely. The metabolic and oxidative stress pathways may participate in PC progression.

## INTRODUCTION

Pancreatic cancer (PC) is a disease that threatens human health and has one of the lowest survival rates; additionally, its annual mortality rate has recently increased among various cancers (from 9^th^ to 7^th^) [1]. Although computed tomography is highly sensitive in the diagnosis of PC [2], its unfavorable prognosis may be attributed to a relatively late diagnosis time. Moreover, recurrence and metastasis are major factors that reduce the survival rate of PC patients [3]. It is important to be able to predict survival in the early stages, and several approaches have been described, including a histologic signature [4], extracellular vesicle long RNA profiling [5], and circulating tumor DNA quantity [6]. Nevertheless, a more holistic and distinguished method is needed for the prognosis prediction in PC patients.

Gene expression-based prognostic signatures have a significant effect on predicting the survival of patients with malignant tumors, such as non-small cell lung cancer [7] and pediatric acute myeloid leukemia [8]. Similarly, the miRNA signature shows predictive value in adults with T-cell lymphoblastic lymphoma [9], and long noncoding RNA shows predictive value in adults with localized clear cell renal cell carcinoma [10]. Most of the existing research has focused on genes for prognosis prediction, whereas pathways based on mRNAs or miRNAs have never been used to predict survival.

Gene set variation analysis (GSVA), a functional enrichment analysis method similar to gene set enrichment analysis (GSEA), allows the assessment of underlying pathway activity variation in each sample by pre-inputting a selected gene set [11]. Recently, differences in pathway activities have been calculated among breast cancer subtypes, and researchers have found that high OSTN-AS1 expression is related to immune-associated pathways using GSVA [12]. This method has excellent performance in identifying prognostic factors for a variety of cancers [13–15]. It can be concluded that as a not widely used method, GSVA may have incredible potential in pathway-related studies.

To the best of our knowledge, for the first time, an approach based on miRNA and mRNA sets was used to predict prognosis in PC patients, and metabolism and oxidative stress were highlighted. More importantly, we found the key components among various pathways that might play an unexpected and significant role in the development of PC. Our results will be helpful for clarifying the intrinsic mechanism of PC progression and provide an essential theoretical basis for presenting new therapeutic approaches.

## RESULTS

### Calculating pathway enrichment scores based on miRNA sets

**Figure 1:**
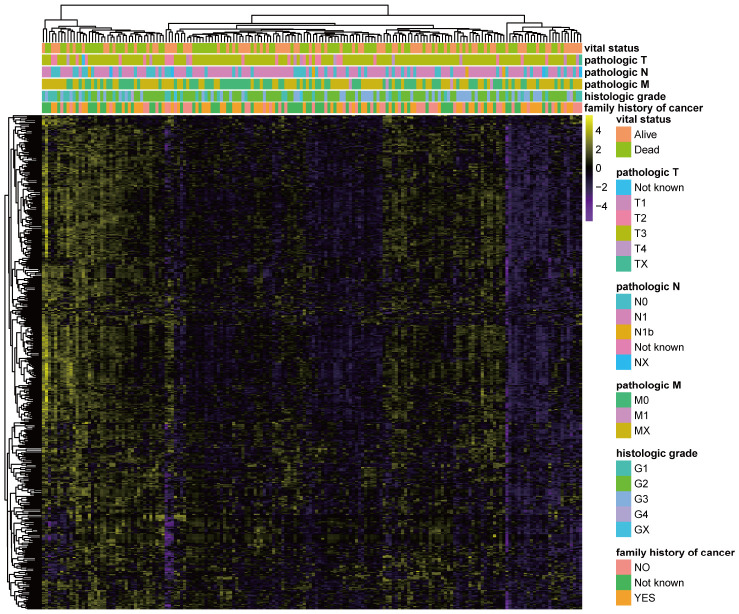
**GSVA results based on miRNA sets in PC. Heatmap of normalized pathway enrichment scores calculated by the GSVA method using the TCGA-PAAD miRNA-seq dataset based on 484 miRNA sets in PC patients with distinct clinical characteristics.** The GSVA method had an ability to calculate enrichment scores of selected gene sets for each patient. Thus, GSVA results containing a matrix of 176 PC patients × 484 miRNA sets with normalized pathway enrichment scores were finally obtained. Heatmap displayed the GSVA results. Each dot represented normalized enrichment score of specific pathway for each patient. The color change represented the level of pathway enrichment scores of every PC patient for every miRNA set-based pathway: purple represented a low score, and yellow represented a high score. Clinical characteristics of each patient were displayed in the top of heatmap, including vital status, pathologic T, pathologic M, pathologic N, histologic grade and family history of cancer.

The GSVA method was used to discriminate pathway expression levels among PC patients. The GSVA method had an ability to calculate enrichment scores of selected gene sets for each patient. Thus, a matrix of patients × gene sets containing pathway enrichment scores would finally obtain through GSVA method. A total of 484 pathways (Supplementary Table 2) were selected based on miRNA sets, including metabolic pathways, signaling pathways, immune-associated pathways, cell function-related pathways, disease-related pathways, and DNA and RNA processing pathways. Normalized pathway enrichment scores of every PC patient for 484 miRNA-set-based pathways were calculated with the GSVA method using the TCGA (The Cancer Genome Atlas)-PAAD (pancreatic adenocarcinoma) miRNA-seq dataset and are displayed in a heatmap (Figure 1). GSVA results containing a matrix of 176 PC patients × 484 miRNA sets with normalized pathway enrichment scores were finally obtained. Heatmap displayed the GSVA results (Figure 1). Pathway enrichment scores based on miRNA sets seemed partially clustered in certain PC patients. Some patients had relatively high pathway enrichment scores, whereas others did not show high expression patterns, suggesting distinct pathway expression maps in individuals with PC, consistent with several published studies [16, 17]. Additionally, by combining clinical parameters, the pathway enrichment score clustered according to different outcomes, including vital status, pathologic T, pathologic M, pathologic N, histologic grade and family history of cancer (Figure 1). These results suggest that specific clinical characteristics might correlate with unique pathway expression profiles.

### Development and validation of the miRNA set-based pathway prognostic signature for PC (miPPSPC)

**Figure 2:**
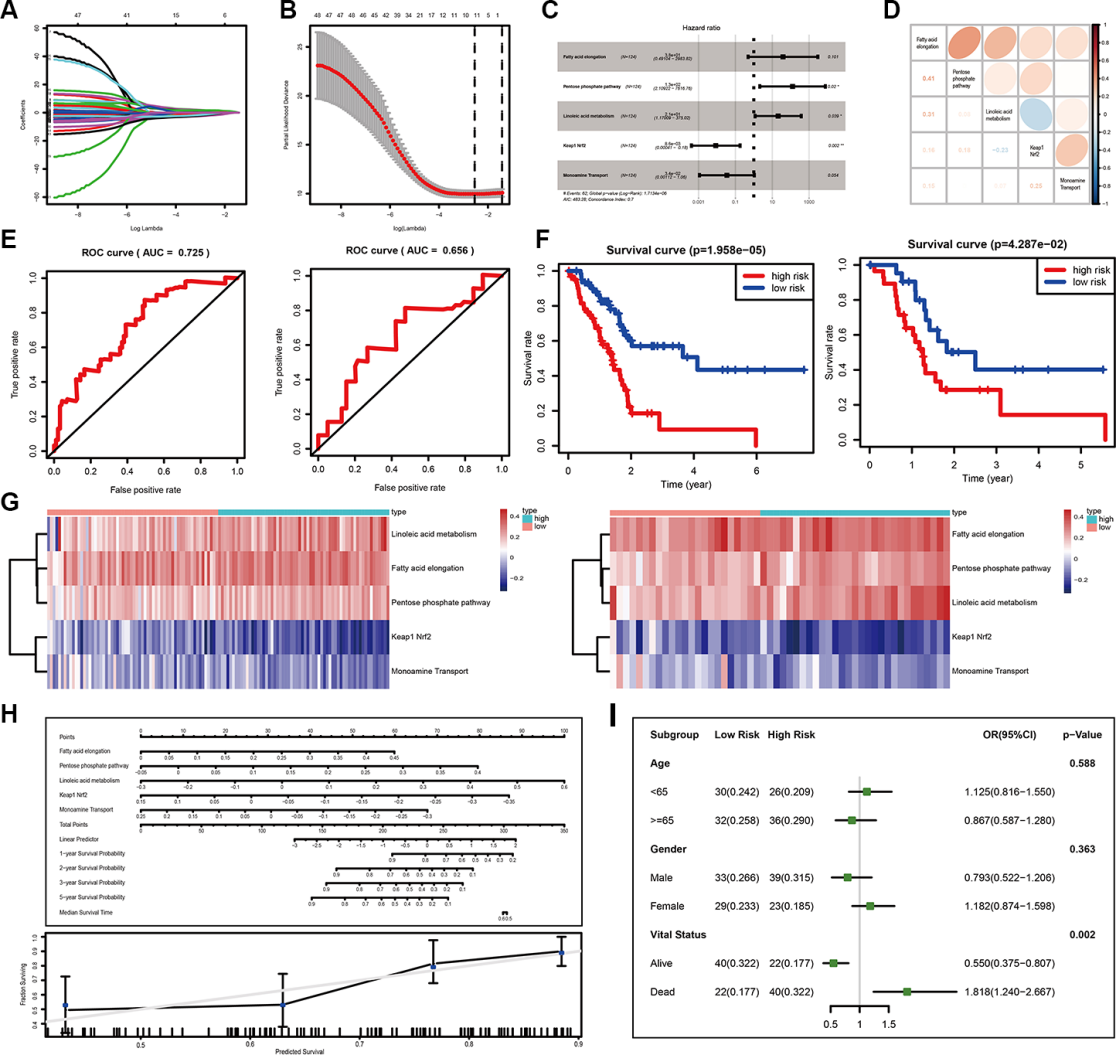
**Development and validation of the miPPSPC.** (**A**, **B**) Least absolute shrinkage and selection operator (LASSO) regression of the pathway enrichment scores of 48 miRNA sets in the training group calculated by the GSVA method. LASSO coefficient profiles of 48 pathways were shown in panel (**A**) and the dotted line indicated the value chosen by tenfold cross-validation. Tenfold cross-validation for tuning parameter selection in the LASSO model was shown in panel (**B**). The partial likelihood deviance was plotted against log (λ), which was the tuning parameter. Partial likelihood deviance values were shown, while error bars represented s.e. The dotted vertical lines were drawn at the optimal values by minimum criteria and 1 - s.e. criteria. In (**A**) and (**B**), the numbers above the graph represent the numbers of pathways involved in the LASSO model. Through the LASSO regression process, 11 of 48 miRNA set-based pathways were filtered for subsequent analysis. (**C**) Forest plot of five pathway-related parameters originating from the Cox proportional hazards model miPPSPC. Through constructing Cox proportional hazards model, 5 of 11 pathways were finally filtered. Unadjusted hazard ratios are shown with 95 percent confidence intervals. AIC, Akaike Information Criterion. (**D**) Correlations among five pathways calculated using pathway enrichment scores of five pathways for PC patients. Red indicated a high correlation, while blue indicated a low correlation. Color depth represented the level of correlation. Correlation coefficients were represented by numbers in the lower left triangle. (**E**) Receiver operating characteristic (ROC) curve of the miPPSPC for the training set (left) and validation set (right). AUC, the area under the curve. (**F**) Kaplan–Meier curves for overall survival by risk score of patients in the training set (left) and validation set (right) based on the miPPSPC. Blue line represented low risk group while red line represented high risk group. Log rank test was used to generate p value. (**G**) Heatmap of the pathway enrichment score distribution of five pathways in the training set (left) and validation set (right). The color change represented the level of pathway enrichment scores of every PC patient for every miRNA set-based pathway: blue represented a low score, and red represented a high score. Patients were divided into low and high risk group according to their risk score. (**H**) Nomogram (upper) of the miPPSPC exhibited a strong correlation among the pathway score, risk score and survival probability. Calibration curve (lower) for the median survival time from the nomogram of the miPPSPC. On the calibration curve, the x-axis represents nomogram-predicted survival, while the y-axis represents observed survival. (**I**) Forest plot of the odds ratio in high-risk and low-risk PC patients based on age, sex and vital status. The risk scores of each patient were calculated based on the miPPSPC, which contains five pathways: fatty acid elongation, the pentose phosphate pathway, linoleic acid metabolism, monoamine transport and Keap1-Nrf2.

Due to the limitation of miRNA-seq datasets of PC, we used TCGA-PAAD miRNA-seq data to develop and validate the miPPSPC. The GSVA results of the TCGA-PAAD miRNA-seq dataset were randomly divided into two parts: seven of ten for the training set and the remainder for the validation set. A total of 124 PC patients were enrolled in the training set, while 52 individuals were enrolled in the validation set. In total, 48 of 484 miRNA set-based pathways were preliminarily filtered using single-factor Cox analysis. We also used least absolute shrinkage and selection operator (LASSO) regression to eliminate collinearity parameters and preliminarily filtered significant miRNA set-based pathways related to survival (Figure 2A, 2B). Through the LASSO regression process, 11 of 48 miRNA set-based pathways were filtered for subsequent Cox proportional hazards model construction. Consequently, a total of 5 miRNA set-based pathways were included in the Cox proportional hazards model miPPSPC: fatty acid elongation (coef= 3.64, HR= 38.27, p= 0.101), the pentose phosphate pathway (coef= 4.84, HR= 126.74, p= 0.020), linoleic acid metabolism (coef= 3.04, HR= 20.94, p= 0.038), monoamine transport (coef= -3.36, HR= 0.03, p= 0.054) and Keap1-Nrf2 (coef= -4.76, HR= 0.008, p= 0.002) (Figure 2C). Four metabolic pathways, namely, fatty acid elongation, the pentose phosphate pathway, linoleic acid metabolism, and monoamine transport, and one oxidative stress pathway, Keap1-Nrf2, were included in the miPPSPC and showed respective correlations (Figure 2D). Harrell’s concordance index (C-index) of the miPPSPC was 0.7 for the training and 0.6 for the validation set. The area under the curve (AUC) of the receiver operating characteristic (ROC) curve of the miPPSPC was 0.725 for the training set and 0.656 for the validation set (Figure 2E). The survival time was shortened in all PC patients with an increasing risk score (Figure 2F). Linoleic acid metabolism, fatty acid elongation and the pentose phosphate pathway were associated with high expression levels in high-risk PC patients in both sets, whereas the oxidative stress pathway Keap1-Nrf2 and monoamine transport were associated with low expression levels (Figure 2G).

Taken together, these results indicate that linoleic acid metabolism, fatty acid elongation and the pentose phosphate pathway negatively influence the prognosis of PC patients. In contrast, patients might benefit from increasing the expression levels of genes involved in the oxidative stress pathway, Keap1-Nrf2, and monoamine transport.

In addition, the performance of the nomogram exhibited feasibility and accuracy when calculating the risk score and predicting the probability of survival (Figure 2H). The associated calibration curve from the nomogram at the median survival time is shown in Figure 2H. We stratified patients by their vital status to determine how the miPPSPC predicts the survival of PC patients regardless of survival time. The risk scores of each patient were calculated based on the miPPSPC containing five pathways: fatty acid elongation, the pentose phosphate pathway, linoleic acid metabolism, monoamine transport and Keap1-Nrf2. The probability of long-term survival decreased with the increasing risk score. PC patients with high risk scores were associated with a low survival rate (Figure 2I). Cohorts of patients <65 years old and females had relatively high risk scores, though the difference was not statistically significant. Taken together, these results confirm the practicability and accuracy of the miPPSPC, which could be a new prognostic tool for PC patients. In addition, we believe that the protumor roles of linoleic acid metabolism, fatty acid elongation, and the pentose phosphate pathway and the antitumor roles of the oxidative stress pathway Keap1-Nrf2 and the monoamine transport pathway will be helpful for illustrating the underlying mechanism and identifying a metabolic therapy for PC patients.

### Optimization of the miPPSPC

**Figure 3:**
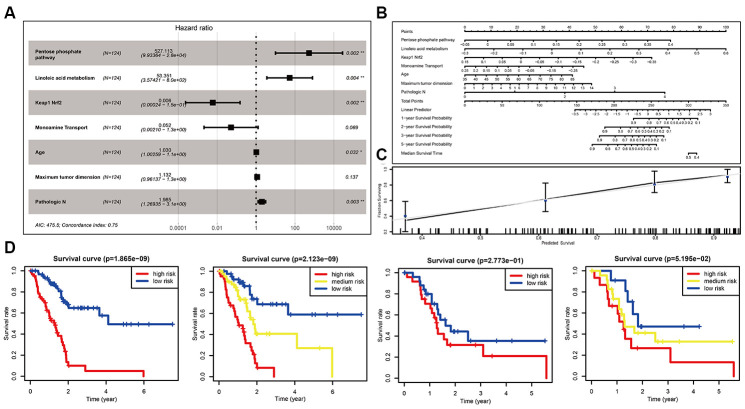
**Optimization of the miPPSPC.** (**A**) Forest plot of four pathways and three clinical indexes originating from the optimized Cox proportional hazards model. Through constructing Cox proportional hazards model, four pathways and three clinical indexes were finally filtered. Unadjusted hazard ratios are shown with 95 percent confidence intervals. AIC, Akaike Information Criterion. (**B**) Nomogram of the optimized miPPSPC that exhibits correlations among the pathway score, clinical indexes, risk score and survival probability. (**C**) Calibration curve for the median survival time from the nomogram of the optimized miPPSPC. On the calibration curve, the x-axis represents nomogram-predicted survival, while the y-axis represents observed survival. (**D**) Kaplan–Meier curves for overall survival by risk score of patients in the training set and validation set based on the optimized miPPSPC. Blue line represented low risk group, yellow line represented high risk group, and red line represented high risk group. Log rank test was used to generate p value.

We hypothesized that the introduction of clinical information would improve the performance of the miPPSPC. When we initially constructed the optimized miPPSPC, we chose all available clinical features of TCGA-PAAD patients, including age, number of lymph nodes, maximum tumor dimension, sex, M stage, N stage and T stage. However, after constructing the Cox proportional hazards model, only three clinical parameters (i.e., age, maximum tumor dimension and pathologic N stage) were used in the optimized m miPPSPC. Finally, a more optimized miPPSPC was established, with a C-index of 0.75 (compared to a C-index of 0.7 for the miPPSPC). Four of the above five pathways were used to construct the optimized miPPSPC through Cox regression: the pentose phosphate pathway, linoleic acid metabolism, monoamine transport and Keap1-Nrf2 (Figure 3A). The results suggested that the above seven factors may together influence clinical outcomes and survival time in PC patients (Figure 3D). The survival time of high-risk PC patients, which was calculated by the optimized miPPSPC, was relatively poor in the training and validation sets. Although the p-value of the validation set did not reach 0.05, it was obvious that the survival curve separated completely between and among subgroups (Figure 3D). Additionally, the performance of the nomogram exhibited feasibility and accuracy when calculating the risk score and predicting the probability of survival (Figure 3B). The associated calibration curve from the nomogram at the median survival time is shown in Figure 3C. These results suggest that compared with the miPPSPC, the optimized miPPSPC might have better performance in the prognosis prediction of PC.

### Effect of the main target genes on the miPPSPC

We wondered whether a portion of the target genes of miRNAs in the miPPSPC wound have significant impacts on tumor progression in PC. Thus, the corresponding target genes of miRNAs in all five pathways were found via miRTarBase, which is an experimentally validated microRNA-target interactions database (Supplementary Table 1). By overlapping all five collections of target genes using a Venn diagram, three genes (CDC34, UVRAG, and SOX9) were found in four pathways, while 10 genes (MYEF2, CCND1, BMI1, E2F1, EP300, RBMXL1, MEIS1, ZNFX1, BIRC3, and TNC) were found in three pathways (Supplementary Figure 1A). A portion of these 13 target genes are transcription factors that regulate the expression of a series of genes, which implied their crucial roles in the transcriptional regulation of PC.

We found that these 13 genes were differentially expressed between tumor and normal tissues in various cancers, especially PC (Supplementary Figure 1A). In addition, a portion of these genes showed a significant influence on the survival of cancer patients (Supplementary Figure 1B). These results suggest that these genes may play key roles in tumor cells via unknown mechanisms. In PC, most genes were found to be associated with the survival status (Supplementary Figure 1C). In addition, four genes had a strong correlation with tumor stage, indicating extremely significant key roles in the progression of PC (Supplementary Figure 1D).

Taken together, these results suggest that five pathways in the miPPSPC influence the prognosis of PC in a critical manner.

### Development and validation of the mRNA set-based pathway prognostic signature for PC (mPPSPC)

**Figure 4:**
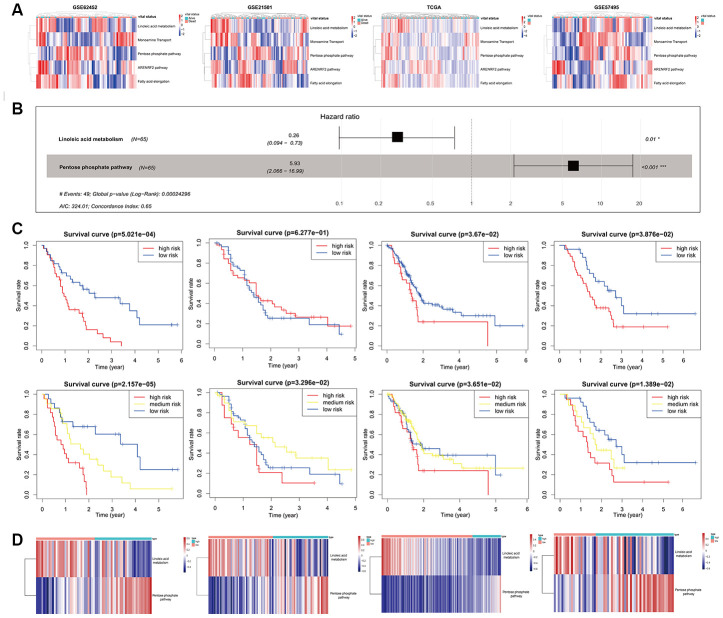
**Development and validation of the mPPSPC.** (**A**) Heatmap of the pathway enrichment score distribution of five mRNA-set-based pathways corresponding to miRNA-set-based pathways in the GSE62452, GSE21501, TCGA-PAAD mRNA-seq and GSE57495 datasets. Pathway enrichment scores were calculated by the GSVA method. PC patients were divided into two groups according to their vital status. The color change represented the level of pathway enrichment scores of every PC patient for every mRNA set-based pathway: blue represented a low score, and red represented a high score. (**B**) Forest plot of two pathways originating from the Cox proportional hazards model mPPSPC. Through constructing Cox proportional hazards model, two pathways were finally filtered. Unadjusted hazard ratios are shown with 95 percent confidence intervals. AIC, Akaike Information Criterion. (**C**) Kaplan–Meier curves for overall survival by risk score of patients in the training set (left two: GSE62452 dataset) and validation set (right six: GSE21501, TCGA-PAAD mRNA-seq and GSE57495 datasets) based on the mPPSPC. Blue line represented low risk group, yellow line represented high risk group, and red line represented high risk group. Log rank test was used to generate p value. (**D**) Heatmap of the pathway enrichment score distribution of two pathways in the mPPSPC for the training set (left one: GSE62452 dataset) and validation set (right three: GSE21501, TCGA-PAAD mRNA-seq and GSE57495 datasets) grouped by low and high risk scores. The color change represented the level of pathway enrichment scores of every PC patient for each mRNA set-based pathway: blue represented a low score, and red represented a high score.

To confirm the possibly pivotal roles of the metabolic pathway and oxidative stress pathway in affecting the prognosis of PC and obtain a more streamlined model, the GSE62452 dataset was used as a training set, whereas the GSE21501, GSE57495 and TCGA-PAAD mRNA-seq datasets were used as validation sets with the help of corresponding pathways based on mRNA sets from the Molecular Signatures Database (MSigDB), including linoleic acid metabolism, the pentose phosphate pathway, the ARENRF2 pathway, monoamine transport, and fatty acid elongation. All the corresponding pathways based on mRNA sets were in line with the five pathways based on miRNA sets obtained in the miPPSPC and the optimized miPPSPC. Pathway enrichment scores of the five pathways based on mRNA sets were calculated using the GSVA method for every PC patient. Diverse pathway enrichment scores between living and deceased PC patients indicated distinct pathway expression patterns in PC patients with diverse prognoses (Figure 4A). Following pathway enrichment score calculation, the Cox proportional hazards model mPPSPC was established using the GSE62452 dataset, with a C-index of 0.65 (Figure 4B). Among the five miRNA-set-based pathways in the miPPSPC and the optimized miPPSPC, two mRNA-set-based pathways were included in the mPPSPC, linoleic acid metabolism and the pentose phosphate pathway, which implied a potentially significant role of metabolic pathways in PC evolution. By validating the mPPSPC with the GSE21501, GSE57495 and TCGA-PAAD mRNA-seq datasets, we found that patients with high risk scores in both the training and validation sets had relatively poorer prognoses (Figure 4C). These results suggest a protumor role of the pentose phosphate pathway and an antitumor role of linoleic acid metabolism in PC, illustrating a diverse and unique mechanism based on mRNA sets (Figure 4D). Together, our findings suggest that metabolic pathways may affect the development of PC and could serve as a prognostic signature to predict the survival of PC patients.

### Effects and relationships of metabolic pathways on the prognosis of PC

**Figure 5:**
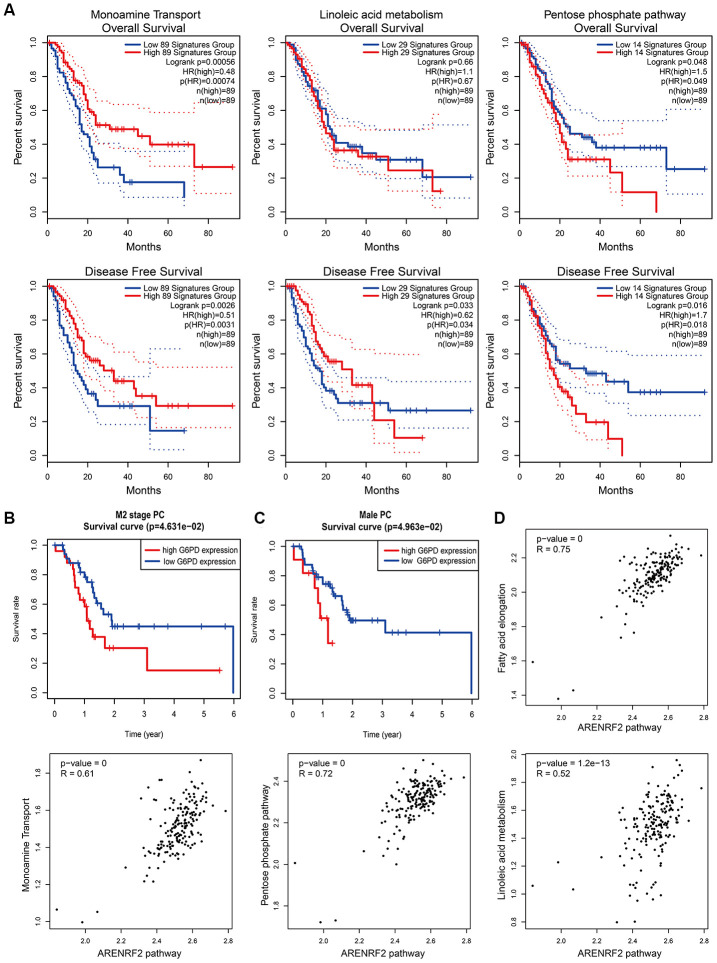
**Effects and relationships of metabolic pathways on the prognosis of PC.** (**A**) Overall survival (upper) and disease-free survival (lower) in PC patients with different expression patterns of monoamine transport (left), linoleic acid metabolism (middle) and the pentose phosphate pathway (right). Low and high expression patterns of pathways were divided by the pathway scores of PC patients calculated by the GEPIA2 tool. Blue line represented low pathway scores group and red line represented high pathway scores group. (**B**, **C**) Kaplan–Meier curves for overall survival of M2 stage (**B**) and male (**C**) PC patients with different G6PD protein expression levels. Protein expression of G6PD in PC was obtained from the TCGA-PAAD reversed-phase protein array. PC patients with incomplete clinical information were excluded. Blue line represented low G6PD expression group and red line represented high G6PD expression group. (**D**) Correlation between metabolic pathways and the oxidative stress pathway in PC patients. Pathway scores were calculated by the GEPIA2 tool according to gene expression in each pathway. Each dot represented specific pathway score of every PC patient.

Subsequently, we examined the impacts of metabolic pathways on the overall survival (OS) and disease-free survival (DFS) of PC patients. Interestingly, both the pentose phosphate pathway and linoleic acid metabolism influenced the survival of PC patients and led to opposite clinical outcomes (Figure 5A), in accordance with the results above (Figure 4D). Additionally, the monoamine transport pathway could prolong the OS and DFS times of PC patients (Figure 5A), consistent with the hypothesis proposed above (Figure 2F). Indeed, the expression of one of the key proteins in the pentose phosphate pathway, G6PD, was related to the survival of M2 stage (Figure 5B) and male (Figure 5C) PC patients according to the TCGA-PAAD reversed-phase protein array (RPPA). High G6PD expression predicted a poor prognosis in M2 stage and male PC patients. In addition, we found a strong correlation between metabolic pathways and the oxidative stress pathway, which suggested a complicated interaction among these processes (Figure 5D). In general, the pentose phosphate pathway and linoleic acid metabolism may significantly influence the development of PC in a previously unseen way.

### Verification of the effects of the pentose phosphate pathway and linoleic acid metabolism in PC using a tissue microarray

**Figure 6:**
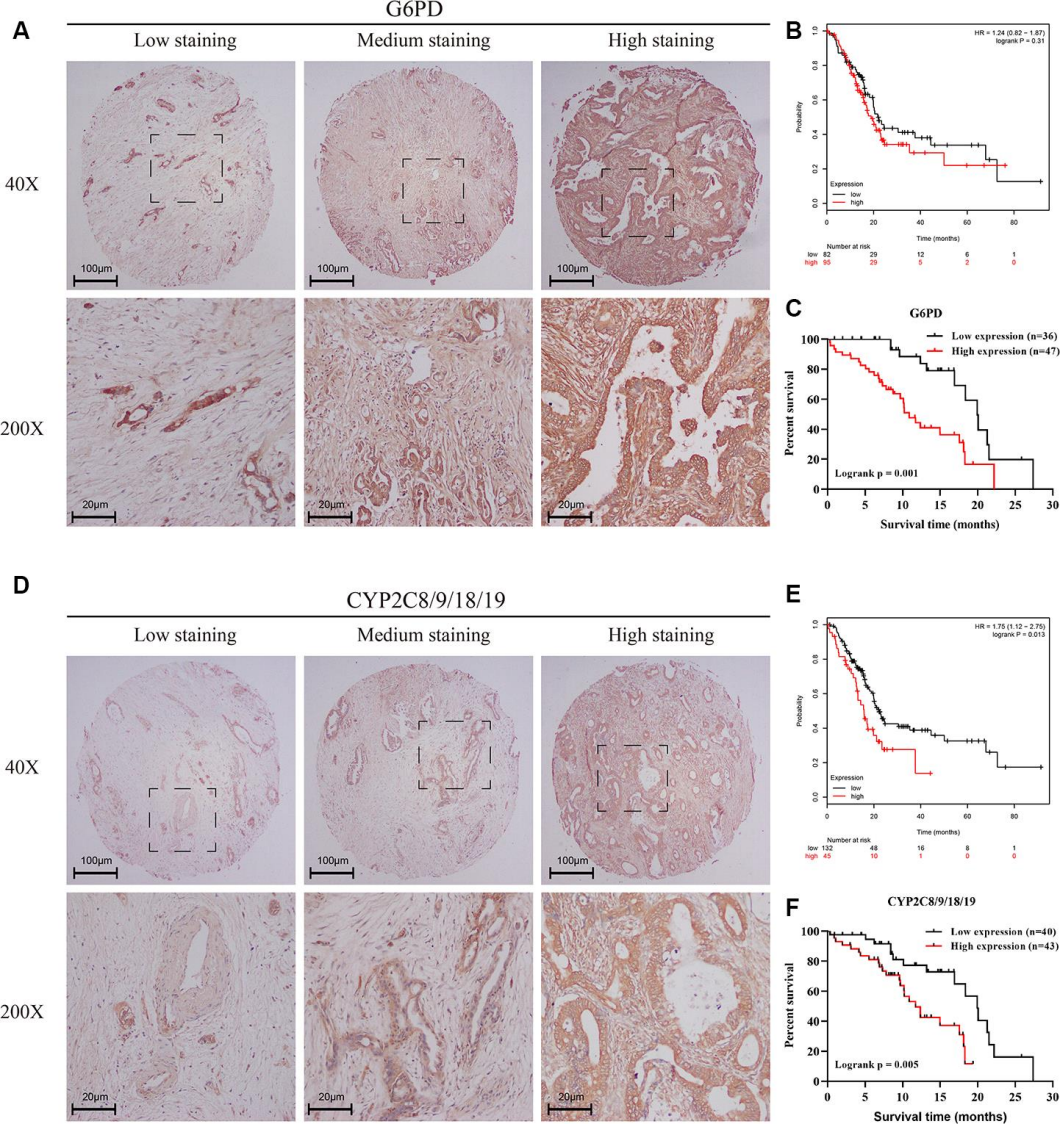
**Verification of the effects of the pentose phosphate pathway and linoleic acid metabolism in PC using a tissue microarray.** (**A**, **D**) IHC staining of a tissue microarray containing 83 PC patients with the G6PD antibody (**A**) or CYP2C8/9/18/19 (**D**) antibody. Intensity was classified as 0, 1+, 2+, and 3+, denoting no, weak, moderate, and strong staining, respectively. The distribution of staining was referred to as the percentage of positive tumor cells (0% to 100%). (**B**, **E**) Kaplan–Meier curves for overall survival of PC patients with different expression levels of G6PD (**B**) and CYP2C8/9/18/19 (**E**) according to the TCGA-PAAD mRNA-seq dataset. Black line represented low expression group and red line represented high expression group. (**C**, **F**) Kaplan–Meier curves for overall survival of PC patients with different expression levels of G6PD (**C**) and CYP2C8/9/18/19 (**F**) according to the IHC results of the tissue microarray containing 83 PC patients. The final G6PD and CYP2C8/9/18/19 expression scores were obtained by multiplying the two variables, intensity and distribution of staining. All samples were further divided into a low expression group and a high expression group according to the expression scores of G6PD (**C**) and CYP2C8/9/18/19 (**F**). Black line represented low expression group and red line represented high expression group.

Finally, we wanted to verify the actual effects of the pentose phosphate pathway and linoleic acid metabolism in PC in our tissue microarray containing 83 PC patients. G6PD serves as a key factor in the pentose phosphate pathway, whereas CYP2C8/9/18/19 serve as key factors in linoleic acid metabolism according to the MSigDB. G6PD (Figure 6A) and CYP2C8/9/18/19 (Figure 6D) show diverse expression patterns depending on the stage of PC, indicating considerable connections between the pentose phosphate pathway and linoleic acid metabolism with clinical stage and pathological grade in PC. Tables 1 and 2 show the clinical characteristics of PC patients stratified according to G6PD and CYP2C8/9/18/19 expression, respectively. G6PD expression seemed to have a significant impact on tumor volume and differentiation, suggesting that the pentose phosphate pathway is involved in the progression of PC. Univariable and multivariable analyses showed that age, clinical stage, G6PD expression and CYP2C8/9/18/19 expression were significantly associated with prognosis in our tissue microarray (Tables 3, 4). Consistent with the RNA-seq data in the TCGA (Figure 6B, 6E), the protein levels of G6PD (Figure 6C) and CYP2C8/9/18/19 (Figure 6F) were significantly associated with the prognosis of PC according to the tissue microarray with immunohistochemical (IHC) staining. Together, these results suggest the essential roles of the pentose phosphate pathway and linoleic acid metabolism pathway in the development of PC, which could influence the survival of PC patients.

**Table 1:** Clinical characteristics of PC patients stratified according to G6PD expression.

**Characteristic**	**High group (n = 47)**	**Low group (n = 36)**	***p*-value**
Age (years)			
< 60 years	14 (29.8)	17 (47.2)	0.162
≥ 60 years	33 (70.2)	19 (52.8)	
Sex			
Female	17 (36.2)	17 (47.2)	0.43
Male	30 (63.8)	19 (52.8)	
Tumor volume (cm^3^)			
<6	20 (42.6)	24 (66.7)	0.05
≥6	27 (57.4)	12 (33.3)	
Differentiation			
Moderate+Well	40 (85.1)	23 (63.9)	0.048
Poor	7 (14.9)	13 (36.1)	
Clinical stage			
I-II	39 (83.0)	34 (94.4)	0.211
III-IV	8 (17.0)	2 (5.6)	
Perineuronal invasion			
No	32 (68.1)	23 (63.9)	0.868
Yes	15 (31.9)	13 (36.1)	

**Table 2:** Clinical characteristics of PC patients stratified according to CYP2C8/9/18/19 expression.

**Characteristic**	**High group (n = 43)**	**Low group (n = 40)**	***p*-value**
Age (years)			
< 60	13 (30.2)	18 (45.0)	0.245
≥ 60	30 (69.8)	22 (55.0)	
Sex			
Female	18 (41.9)	16 (40.0)	1
Male	25 (58.1)	24 (60.0)	
Tumor volume (cm^3^)		
<6	22 (51.2)	22 (55.0)	0.897
≥6	21 (48.8)	18 (45.0)	
Differentiation			
Moderate+Well	34 (79.1)	29 (72.5)	0.658
Poor	9 (20.9)	11 (27.5)	
Clinical stage			
I-II	37 (86.0)	36 (90.0)	0.829
III-IV	6 (14.0)	4 (10.0)	
Perineuronal invasion		
No	30 (69.8)	25 (62.5)	0.64
Yes	13 (30.2)	15 (37.5)	

**Table 3:** Cox univariable and multivariable analyses of clinicopathological variables and G6PD expression in relation to OS in PC patients.

**Clinical factor**	**Univariable analysis**				**Multivariable analysis**		
	**HR**	**95% CI**	***p*-value**		**HR**	**95% CI**	***p*-value**
Age (≥ 60 vs. < 60 years)	2.088	1.044-4.178	0.037		2.046	1.002-4.178	0.049
Sex (Male vs. Female)	1.522	0.764-3.033	0.232		-	-	-
Differentiation (Poor vs. Moderate+Well)	1.442	0.711-2.922	0.31		-	-	-
Tumor volume (≥6 vs. <6 cm^3^)	1.902	0.995-3.636	0.052		-	-	-
Perineuronal invasion (Positive vs. Negative)	1	0.513-1.951	0.999		-	-	-
Clinical stage (III-IV vs. I-II)	3.546	1.512-8.315	0.004		2.977	1.256-7.059	0.013
G6PD expression (High vs. Low)	3.411	1.657-7.019	0.001		3.222	1.527-6.8	0.002

**Table 4:** Cox univariable and multivariable analyses of clinicopathological variables and CYP2C8/9/18/19 expression in relation to OS in PC patients.

**Clinical factor**	**Univariable analysis**				**Multivariable analysis**		
	**HR**	**95% CI**	***p*-value**		**HR**	**95% CI**	***p*-value**
Age (≥ 60 vs. < 60 years)	2.088	1.044-4.178	0.037		2.046	1.002-4.178	0.049
Sex (Male vs. Female)	1.522	0.764-3.033	0.232		-	-	-
Differentiation (Poor vs. Moderate+Well)	1.442	0.711-2.922	0.31		-	-	-
Tumor volume (≥6 vs. <6 cm^3^)	1.902	0.995-3.636	0.052		-	-	-
Perineuronal invasion (Positive vs. Negative)	1	0.513-1.951	0.999		-	-	-
Clinical stage (III-IV vs. I-II)	3.546	1.512-8.315	0.004		3.393	1.443-7.976	0.005
CYP2C8/9/18/19 expression (High vs. Low)	2.983	1.4-6.354	0.005		2.915	1.366-6.218	0.006

## DISCUSSION

Many people suffer from a variety of cancers, including PC, with the highest mortality rate [18]. More effective and accurate methods for predicting the prognosis of PC are urgently needed. In this research, novel pathway prognostic signatures, including the miPPSPC, optimized miPPSPC and mPPSPC, were proposed with the help of GSVA and are based on miRNA sets (miPPSPC and optimized miPPSPC) and mRNA sets (mPPSPC). Our models innovatively showed excellent performance in predicting the survival of PC patients. Moreover, a collection of pathways (four metabolic pathways: fatty acid elongation, the pentose phosphate pathway, linoleic acid metabolism, and monoamine transport; and one oxidative stress pathway: Keap1-Nrf2), especially the pentose phosphate pathway and linoleic acid metabolism, likely dominate the progression of PC. This is the first study to investigate a tumor prognostic signature from a holistic pathway-related perspective. These results may provide a new research direction for unveiling the underlying mechanism of PC.

miRNAs are a class of small, endogenous single-stranded noncoding regulatory RNAs of approximately 22 nucleotides in length initially discovered in 1993 [19]. Studies over the past two decades have forged links between the dysregulation of miRNAs and different types of cancer [20]. In addition to miRNA analyses (e.g., GSEA and gene regulatory network analysis), microRNA enrichment analyses of functions and signaling pathways in cancer have also been performed [21]. There are several relationships between miRNAs and pathways. Metabolic reprogramming is a hallmark of cancer. miRNAs have been perceived to be relevant to cancer-related metabolic pathways, including fatty acid metabolism, the pentose phosphate pathway, the tricarboxylic acid cycle, glycolysis, amino acid metabolism, and other metabolism-related oncogenic signaling pathways [22]. Our miRNA set-based pathway prognostic signature is based directly on the potential network regulation of miRNAs to pathways.

Pathway activity plays a significant role in tumor growth, apoptosis, metastasis, recurrence, etc. Targeting metabolic intermediates was considered effective in a phase I study in cancer [23]. We identified four metabolic pathways, namely, fatty acid elongation, the pentose phosphate pathway, linoleic acid metabolism, and monoamine transport, and one oxidative stress pathway, Keap1-Nrf2, among a large number of miRNA set-based pathways and confirmed their significance using a mRNA set-based pathway model. Metabolic pathways affect nutrient cycling in humans and are believed to alter the features of cancer cells. The kynurenine metabolic axis has been reported to be related to PC and has the ability to enhance aggressiveness and influence outcomes [24]. Fatty acid elongation plays a key role in human nonalcoholic steatohepatitis development [25]. Human NASH-related hepatocellular carcinoma is associated with increased levels of fatty acid elongation [26]. The pentose phosphate pathway participates in the biosynthesis of ribonucleotide precursors and NADPH [27] and has been found to be involved in tumor progression. Research reports that Rev-erbα, a nuclear receptor, inhibits gastric cancer cell proliferation by inhibiting the pentose phosphate pathway [28]. Similarly, pentose phosphate pathway blockade induces reactive oxygen species (ROS)-mediated apoptosis in thyroid cancer cells [29]. According to proteomic analysis, the pentose phosphate pathway is activated in PC stem cells [30]. Emerging evidence suggests that G6PD, a gatekeeper of the pentose phosphate pathway, is a potential therapeutic target in human cancers [31]. Linoleic acid metabolism plays a functional role in cancer processes such as cell growth, cell survival, angiogenesis, cell invasion, metastatic potential and immunomodulation [32]. For instance, THF diols regulate cell proliferation by modulating specific enzymatic sites involved in linoleic acid metabolism in human breast cancer cells [33]. The Keap1-Nrf2 pathway is a regulator of cytoprotective responses to endogenous and exogenous stresses induced by ROS, which are an unenviable part of aerobic life, and its dysregulation is observed in cancer cells [34, 35]. Synchronously, certain sensors, such as Keap1/Nrf2, HIF-1α, NF-kB and other regulatory pathways, exert a coordinated function in human biology and pathology related to the realization of ROS effects [36].

Among the various pathways associated with tumor progression, metabolic pathway activity could affect the level of metabolites. The pentose phosphate pathway is a basic component of cellular metabolism and is important for maintaining carbon homeostasis; it also provides precursors for nucleotide and amino acid biosynthesis, reduces molecules for anabolism, and defeats oxidative stress [37]. Pentose phosphate pathway flux is increased in human cancer and influences cancer progression [31]. In addition, linoleic acid metabolism may be related to increased carcinogenesis and enhanced tumor progression [38]. However, whether these two pathways are significant in the development of PC remains unclear.

In our research, we established a three-pathway prognostic signature based on mRNA sets, miRNA sets or the clinical features of PC patients: the miPPSPC, optimized miPPSPC, and mPPSPC. Among these prognostic signatures, the C-index of the optimized miPPSPC reached 0.75, indicating better prognostic efficiency than the miPPSPC and mPPSPC. Although the efficiency of the mPPSPC was roughly similar to that of the miPPSPC, the mPPSPC contained only two pathway parameters compared with five pathway parameters in the miPPSPC, suggesting that the mPPSPC makes survival prediction simpler. In addition, miRNAs function by regulating mRNA expression, and the latter function by directly translating proteins, suggesting that the mPPSPC might have more direct prediction effects than the miPPSPC. For instance, we obtained both the pentose phosphate pathway and linoleic acid metabolism with the mPPSPC; these pathways were also identified by the miPPSPC. We could then directly investigate the influences of the expression levels of both pathways on the progression of PC.

In summary, we proposed unique prognosis prediction models based on miRNA sets and mRNA sets that showed excellent ability in estimating the survival of PC patients. According to the pathway prognostic signatures, four metabolic pathways, namely, fatty acid elongation, the pentose phosphate pathway, linoleic acid metabolism, and monoamine transport, and one oxidative stress pathway, Keap1-Nrf2, were identified as potentially important factors that influence the prognosis of PC. This study is the first to filter and verify prognosis-related factors from the perspective of pathways in a more integrated and comprehensive manner. Our results will be helpful for survival prediction and studies on the intrinsic mechanism of PC.

## MATERIALS AND METHODS

### Data acquisition and processing

The datasets used in this paper were retrospectively collected from The Cancer Genome Atlas (TCGA) and Gene Expression Omnibus (GEO). RNA-seq FPKM data and relevant clinical records of the TCGA-PAAD cohort were downloaded from UCSC Xena (https://xenabrowser.net/datapages/). Microarray data and corresponding follow-up information of PC patients were obtained from the GEO repository (https://www.ncbi.nlm.nih.gov/geo/) (GSE62452, GSE21501 and GSE57495). All GEO datasets were normalized using the RMA method or global normalization. Adjacent nontumor tissue samples were removed, and all patients with incomplete prognostic information were excluded. The protein expression of G6PD in PC was obtained from the TCGA-PAAD RPPA.

### Retrieval of pathway-related miRNA sets and mRNA sets and pathway enrichment analysis using GSVA

miRNA sets were downloaded from the miEAA (https://ccb-compute2.cs.uni-saarland.de/mieaa_tool/). The miRWalk Pathways miRNA file, which contains a total of 484 miRNA sets, was selected as the target miRNA set for further analysis (Supplementary Table 2). mRNA sets including linoleic acid metabolism, the pentose phosphate pathway, the ARENRF2 pathway, monoamine transport, and fatty acid elongation were acquired from the MSigDB (http://software.broadinstitute.org/gsea/index.jsp) (Supplementary Table 3). All miRNA sets and mRNA sets were transformed to the corresponding format awaiting processing using the R *GSVAdata* package. The *GSVA* package was performed in R 3.6.1 to calculate the enrichment score of the pathways in each sample. miRNA sets were used to obtain scores for the TCGA-PAAD miRNA-seq dataset, whereas mRNA sets were used to obtain scores for the TCGA-PAAD mRNA-seq, GSE62452, GSE21501 and GSE57495 datasets.

### Establishment and validation of the miPPSPC

The GSVA results of the TCGA-PAAD miRNA-seq dataset were randomly divided into two parts: seven of ten for the training set and the remainder for the validation set. In total, 48 of 484 miRNA set-based pathways were preliminarily filtered using single-factor Cox analysis. LASSO regression was used for collinearity parameter elimination using the glmnet package. During LASSO regression, the enrichment scores of a total of 48 miRNA set-based pathways for PC patients were input. After LASSO regression, 11 of 48 miRNA set-based pathways were filtered. Subsequently, a Cox proportional hazards model was used to filter and assess their association with OS among 11 miRNA set-based pathways. Cox regression was performed using the survival and survminer R packages with data on the enrichment scores of 11 miRNA set-based pathways filtered from LASSO regression for PC. Then, we established the miPPSPC in the training set and validated it in the validation set. The optimized miPPSPC was established by adding clinical information on TCGA-PAAD patients.

### Establishment and validation of the mPPSPC

According to the miPPSPC, five corresponding mRNA sets from the MSigDB were used to construct the mPPSPC: linoleic acid metabolism, the pentose phosphate pathway, the ARENRF2 pathway, monoamine transport, and fatty acid elongation. The GSE62452 dataset was used to establish the mPPSPC, whereas the GSE21501, GSE57495 and TCGA-PAAD mRNA-seq datasets were used to validate the mPPSPC.

### IHC staining of the tissue microarray and evaluation of reaction intensity

Paraffin sections of PC tissue microarrays containing 83 PC patients were used for IHC staining. Sections were stained with primary antibodies against G6PD (1:50, Proteintech Group, Chicago, IL, USA) and CYP2C8/9/18/19 (1:100, Proteintech Group, Chicago, IL, USA) according to the product manual after routine steps. Phosphate-buffered saline (PBS) was used as a control.

The scoring of positive immunoreactivity was performed as described previously [39]. Intensity was classified as 0, 1+, 2+, and 3+, denoting no, weak, moderate, and strong staining, respectively. The distribution of staining was referred to as the percentage of positive tumor cells (0% to 100%). The final G6PD and CYP2C8/9/18/19 expression scores were obtained by multiplying the two variables together. Additionally, all samples were further divided into a low expression group and a high expression group according to the expression scores of G6PD and CYP2C8/9/18/19.

### Statistical analysis

Statistical analysis and visualization were performed using R 3.6.1 (https://www.r-project.org/) with the survivalROC, pheatmap, foreign, and corrplot packages. Survival curves were drawn and compared between subgroups using the survival package. Furthermore, GEPIA2 (http://gepia2.cancer-pku.cn/#index) and Kaplan-Meier plotter (http://kmplot.com/analysis/) were used for additional survival and correlation analyses. The nomogram was generated using the rms package. The Venn diagram was drawn using the webtool (http://bioinformatics.psb.ugent.be/webtools/Venn/).

### Ethics approval

All the procedures involving human tumor biopsies were performed with the approval of the Ethics Committee of the First Affiliated Hospital of Third Military Medical University, PLA.

## Supplementary Material

Supplementary Figure 1

Supplementary Table 1

Supplementary Table 2

Supplementary Table 3
