# Supplementary material for: Establishing and validating a pathway prognostic signature in pancreatic cancer based on miRNA and mRNA sets using GSVA
Source: Aging (Albany NY). 2020 Nov 10;12(22):22840–58. doi: 10.18632/aging.103965 (PMC7746356; doi:10.18632/aging.103965)
Supplement: Supplementary Figure 1 [file aging-12-103965-s001..pdf]

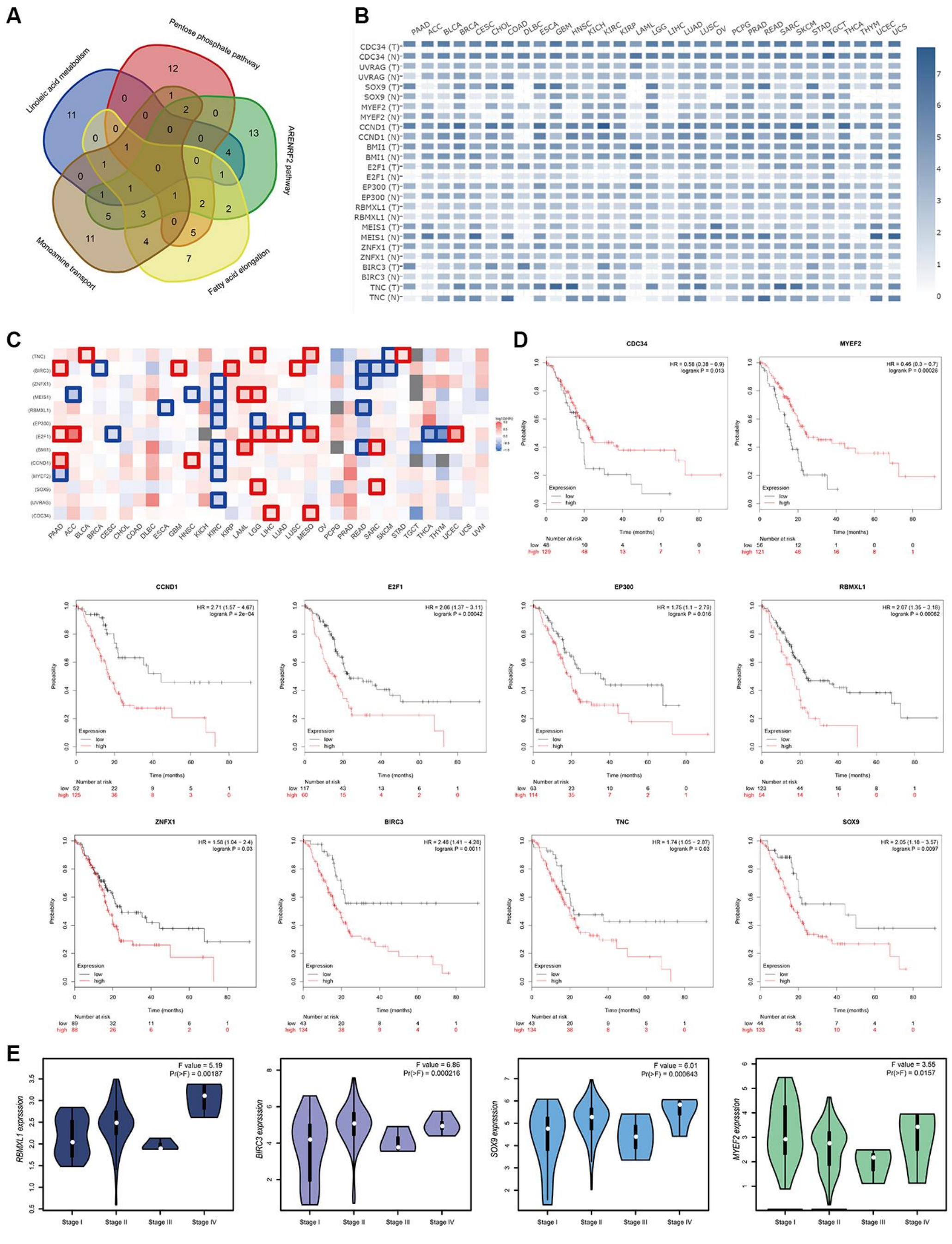

Supplementary Figure 1. Overlapping target genes in the miRNA-based pathway examination. (**A**) Venn diagram of overlapping target genes in five miRNA-based pathways in the miPPSPC. (**B**) Expression of the main target genes in different cancers. The color change represented the level of gene expression for cancer patients in TCGA: blue represented high gene expression level while white represented low gene expression level. (**C**) Survival map of main target genes on different cancers in TCGA generated by GEPIA2 tool. Survival map could compare the survival contribution of multiple genes in multiple cancer types, estimated using Mantel–Cox test. Significant results were framed in red or blue, representing cancer promoting or inhibiting effects, respectively. (**D**) Kaplan–Meier curves for overall survival of PC patients in TCGA with different gene expression levels were shown. Black line represented low gene expression group and red line represented high gene expression group. (**E**) Expression of four main target genes in PC patients with different stages.
