# Supplementary material for: Establishing and validating a pathway prognostic signature in pancreatic cancer based on miRNA and mRNA sets using GSVA
Source: Aging (Albany NY). 2020 Nov 10;12(22):22840–58. doi: 10.18632/aging.103965 (PMC7746356; doi:10.18632/aging.103965)
Supplement: Supplementary Table 1 [file aging-12-103965-s002..pdf]

SUPPLEMENTARY TABLE

Supplementary Table 1. Target genes of five pathways in miPPSPC.

Fatty acid elongation	Target	Pentose phosphate pathway	Target	Linoleic acid metabolism	Target	Keap1 Nrf2	Target	Monoamine Transport	Target
hsa-miR-1229-3p	VWA8	hsa-miR-671-5p	CDR1	hsa-miR-132-3p	SIRT1	hsa-miR-24-2-5p	BCL2	hsa-miR-125b-5p	BMPR1B
hsa-miR-152-3p	HLA-G	hsa-miR-1229-3p	VWA8	hsa-miR-9-5p	MMP13	hsa-miR-1-3p	MYEF2	hsa-miR-197-3p	TSPAN3
hsa-miR-1-3p	MYEF2	hsa-miR-149-5p	SP1	hsa-miR-128-3p	NTRK3	hsa-miR-877-5p	RPLP1	hsa-miR-203a-3p	UVRAG
hsa-miR-215-5p	WNK1	hsa-miR-1-3p	MYEF2	hsa-miR-146b-5p	NFKB1	hsa-miR-33a-5p	ABCA1	hsa-miR-215-5p	WNK1
hsa-miR-877-5p	RPLP1	hsa-let-7e-5p	HMGA2	hsa-miR-92b-3p	SLC15A1	hsa-miR-16-5p	BMI1	hsa-miR-130b-3p	TP53INP1
hsa-miR-151a-3p	ZNF763	hsa-miR-15b-5p	CCNE1	hsa-miR-488-3p	POMC	hsa-miR-153-3p	BCL2	hsa-miR-141-3p	ZEB2
hsa-miR-877-3p	SLC6A8	hsa-miR-296-3p	KCNH1	hsa-miR-124-3p	SOX9	hsa-miR-10a-5p	HOXA1	hsa-miR-30c-5p	MUC17
hsa-miR-16-5p	BMI1	hsa-miR-193b-3p	CCND1	hsa-miR-10a-5p	HOXA1	hsa-miR-24-3p	FEN1	hsa-miR-24-3p	FEN1
hsa-miR-15b-5p	CCNE1	hsa-miR-760	CSNK2A1	hsa-miR-338-3p	UBE2Q1	hsa-miR-365a-3p	CCND1	hsa-miR-92a-3p	UVRAG
hsa-miR-99a-5p	RAVER2	hsa-miR-484	FIS1	hsa-miR-98-5p	E2F1	hsa-miR-744-5p	ARL15	hsa-miR-26b-5p	EP300
hsa-miR-193b-3p	CCND1	hsa-miR-935	PURB	hsa-miR-15a-5p	BMI1	hsa-miR-193b-3p	CCND1	hsa-miR-204-5p	MEIS1
hsa-miR-26b-5p	EP300	hsa-miR-331-3p	ERBB2	hsa-miR-27b-3p	NOTCH1	hsa-miR-26b-5p	EP300	hsa-miR-421	RBMXL1
hsa-miR-484	FIS1	hsa-miR-1180-3p	FAM200B	hsa-miR-145-5p	BNIP3	hsa-miR-15a-5p	BMI1	hsa-miR-21-5p	RASGRP1
hsa-miR-615-3p	LCOR	hsa-miR-92a-3p	UVRAG	hsa-miR-449c-5p	MYC	hsa-miR-505-3p	SRSF1	hsa-miR-7-5p	SNCA
hsa-miR-331-3p	ERBB2	hsa-miR-1296-5p	MCM2	hsa-miR-30a-5p	BDNF	hsa-miR-214-3p	EZH2	hsa-miR-34a-5p	BIRC3
hsa-miR-92a-3p	UVRAG	hsa-miR-346	EFEMP2	hsa-miR-488-5p	AR	hsa-miR-142-5p	NFE2L2	hsa-miR-221-3p	CDKN1B
hsa-miR-29a-3p	CDK6	hsa-miR-34a-5p	BIRC3	hsa-miR-335-5p	TNC	hsa-miR-196a-5p	SPRR2C	hsa-miR-296-3p	KCNH1
hsa-miR-29c-3p	COL3A1	hsa-miR-92b-5p	KIAA1671	hsa-let-7b-5p	CDC34	hsa-miR-140-3p	NRIP1	hsa-miR-17-5p	ZNFX1
hsa-let-7f-1-3p	MECR	hsa-miR-17-5p	ZNFX1	hsa-miR-107	PLAG1	hsa-miR-92a-3p	UVRAG	hsa-let-7a-5p	CDK6
hsa-miR-186-5p	FOXO1	hsa-let-7b-5p	CDC34	hsa-miR-103a-3p	GPD1	hsa-miR-122-5p	CYP7A1	hsa-let-7b-5p	CDC34
hsa-let-7b-5p	CDC34	hsa-miR-181a-5p	NLK			hsa-miR-7-5p	SNCA	hsa-miR-98-5p	E2F1
hsa-miR-98-5p	E2F1	hsa-miR-20a-3p	YTHDC1			hsa-miR-148b-3p	HLA-G	hsa-miR-200a-3p	DLX5
hsa-miR-339-5p	BCL6	hsa-miR-378a-5p	SUFU			hsa-miR-125a-5p	CDKN1A	hsa-miR-124-3p	SOX9
hsa-miR-124-3p	SOX9	hsa-miR-339-5p	BCL6			hsa-miR-34a-5p	BIRC3	hsa-miR-106b-5p	ITCH
hsa-miR-192-5p	CLIC1					hsa-miR-421	RBMXL1	hsa-miR-192-5p	CLIC1
hsa-miR-155-5p	MEIS1					hsa-miR-423-5p	RABAC1	hsa-miR-20a-5p	HIF1A
hsa-miR-320a	POLR3D					hsa-miR-17-5p	ZNFX1	hsa-miR-744-5p	ARL15
hsa-miR-421	RBMXL1					hsa-miR-144-3p	PLAG1	hsa-miR-23a-3p	CXCL12
						hsa-miR-92b-3p	SLC15A1	hsa-miR-335-5p	TNC
						hsa-miR-128-3p	NTRK3	hsa-miR-320a	POLR3D
						hsa-miR-17-3p	ICAM1	hsa-miR-425-5p	ZNF700
						hsa-miR-200a-3p	DLX5	hsa-miR-375	TIMM8A
						hsa-miR-124-3p	SOX9		
						hsa-miR-106b-3p	SSB		
						hsa-miR-27a-3p	RUNX1		
						hsa-miR-155-5p	MEIS1		
						hsa-miR-335-5p	TNC		
						hsa-miR-375	TIMM8A		
